# Psychological Outcomes and Associated Factors Among the International Students Living in China During the COVID-19 Pandemic

**DOI:** 10.3389/fpsyt.2021.707342

**Published:** 2021-08-13

**Authors:** Md. Dhedharul Alam, Jing Lu, Li Ni, Shaohua Hu, Yi Xu

**Affiliations:** ^1^Department of Psychiatry, The First Affiliated Hospital, Zhejiang University School of Medicine, Hangzhou, China; ^2^The Key Laboratory of Mental Disorder Management in Zhejiang Province, Hangzhou, China; ^3^Department of Psychiatry, Fuyang Third Peoples Hospital, Hangzhou, China

**Keywords:** China, COVID-19, international students, mental health, psychological outcomes

## Abstract

**Background:** The emergence of coronavirus disease 2019 (COVID-19) has created a severe mental health problem for international students living in China. Despite the little information on the psychological impact on international students, we aimed to assess the psychological outcomes and associated factors among international students currently living in China during the COVID-19 pandemic.

**Methods:** An online cross-sectional survey was conducted from May 28, 2020 to June 12, 2020 on 402 full-time international students across 26 provinces in China. The frequency of symptoms of depression, anxiety, stress, insomnia, psychological distress, loneliness, and fear was assessed with the English versions of the Depression Anxiety Stress Scale (DASS-21), Insomnia Severity Index (ISI), Kessler Psychological Distress Scale (K6), University of California, Los Angeles, Loneliness Scale (UCLA-LS), and Fear of COVID-19 scale (FCV-19S) scales, respectively.

**Results:** The prevalence of symptoms of depression (73.4%), anxiety (76.6%), stress (58.5%), insomnia (77.6%), psychological distress (71.4%), loneliness (62.4%), and fear (73.1%) among international students during the COVID-19 pandemic was shown. The prevalence of moderate to extremely severe symptoms of all psychological outcomes was significantly associated with 26–30-year-old students, students who lived with roommates, and students who stayed in China shorter than 2 years. Participants in the central region reported significantly moderate to extremely severe symptom levels of all the psychological outcomes except fear symptoms. Univariate analysis indicated that a significant association of all psychological outcomes was found among 26–30-year-old students and students who stayed in China shorter than 2 years. Multivariate analysis showed that Engineering, Business, Social Sciences and Law, and Language students were significantly associated with the symptoms of depression, anxiety, insomnia, and fear. Participants staying in China for shorter than 2 years were associated with a higher risk of all psychological outcomes except psychological distress and loneliness symptoms.

**Conclusions:** We found a higher prevalence of psychological outcomes and risk factors among international students during the COVID-19 pandemic. We immediately appealed to university authorities, mental health professionals, and government officials to provide mental health interventions and strategies for their international students, particularly young, central region students, living with roommates, different study backgrounds, and short time staying during the pandemic.

## Introduction

A large number of studies have established that any stressful event such as natural disasters and manufactured traumas has a significant mental health impact among affected individuals (1, 2). Recently, such diseases, namely, the novel coronavirus, have come out in China. The first four cases were reported on December 29, 2019, and all were linked to the Huanan (Southern China) Seafood Wholesale Market. All four patients were identified by local hospitals using a surveillance mechanism for “Pneumonia of unknown etiology” in Wuhan, the capital city of Hubei province, central region, China (3). The local hospital identified the coronavirus on January 7, 2020, and named it severe acute respiratory syndrome coronavirus 2 (SARS-CoV-2) (3). On January 30 of the same year, the World Health Organization declared the outbreak of a Public Health Emergency of International Concern (PHEIC) (4), the official name of the new disease was named coronavirus disease 2019 (COVID-19) on February 11, 2020 (5). It was officially declared as a global pandemic on March 11, 2020 (6).

As of June 13, 2020, the official website of the National Health Commission of China confirmed that 83,075 cases of COVID-19 had been identified, while 4,634 people have died of COVID-19 across China (7). The COVID-19 pandemic has impacted 216 countries, areas, or territories globally and infected 7,553,182 people, including 423,349 deaths documented globally by the last count of June 13, 2020 (8). To point out this serious issue, the World Health Organization proclaimed that there would be high possibilities of an increase in stress, anxiety, fear, behavioral changes, loneliness, depression, and suicidal activities due to the COVID-19 pandemic (9). A recent review showed that high rates of indications of anxiety (6.33–50.9%), depression (14.6–48.3%), post-traumatic stress disorder (7–53.8%), psychological distress (34.43–38%), and stress (8.1–81.9%) were reported among general population during the COVID-19 outbreak in China, Spain, Italy, Iran, the USA, Turkey, Nepal, and Denmark (10).

It was an assumption that by 2020, the volume of international students would soar up to 8 million globally (11). On April 15, 2019, the Ministry of Education of the People's Republic of China announced that nearly 500,000 international students are currently studying in China (12). During the winter vacation and spring festival holiday, few international students went back to their own countries. However, there was a considerable number of international students who did not go back and decided to keep staying in China. The university authorities advised the students to stay and not leave the campus to ensure the health and safety of all international students. This situation hampered their studies, interrupted their daily routines and habits, and severely impacted their physical and mental health. Already many countries focused on the psychological impact of the COVID-19 epidemic on the universities' local students (13–16). However, compared to local students under regular circumstances, international students are more prone to mental health problems (17).

At present, to the best of our knowledge, this is the first study to investigate the magnitude of psychological outcomes and associated factors by using standardized rating scales among the international students living in China during the COVID-19 outbreak. Not only during the COVID-19 outbreak but also during previous bio-disasters that there had been less information about the mental health status among international students around the world (18–20). Hence, this study aimed to evaluate the psychological outcomes among international students who remained in China during the COVID-19 epidemic period by quantifying the magnitude of depression, anxiety, stress, insomnia, psychological distress, loneliness, and fear by analyzing potential risk factors associated with these symptoms.

## Materials and Methods

### Study Design

This online cross-sectional survey study was conducted through a snowball sampling process *via* WeChat from May 28, 2020, to June 12, 2020. We developed an online questionnaire using Questionnaire Network (https://www.wenjuan.com/), the link to which could be shared *via* WeChat (a popular Chinese social media platform). Clicking the survey link in WeChat took international students directly to the online questionnaire. We urged these international students to share the survey link to their WeChat contact list friends and friends they considered suitable for this survey. The snowball sampling process continued until a sufficient number of sample sizes were obtained. Participants anonymously completed the self-administered electronic questionnaire for ~20 min with no financial incentive. The participants were fully informed that they were free to discontinue participation at any time, and the researcher guarantees the confidentiality of participants' information. Overall, data were collected from 84 universities and 26 provinces across seven geographical regions of China (Eastern region, Northern region, Southwest region, Northeast region, Central region, Southern region, and Northwest region). Out of these seven geographical regions of China, international students from 45 countries filled out the online questionnaire. These 45 countries are divided into four geographical areas (Asia, Africa, Europe, and America). Additionally, the Ethics Committee of the First Affiliated Hospital, Zhejiang University School of Medicine, approved this study. Respondents received an online written informed consent form before answering the questionnaire.

### Participants

The target sample size of the participants was determined using the formula:

*n* = [z2 × p × (1 – p)/e2]/[1 + (z2 × p × (1 – p)/(e2 × N))]
where *z* = 1.96 for a confidence level (α) of 95%,
*p* = 0.5 for proportion (expressed as a decimal),
*e* = 5% for margin of error, and
*N* = 500,000 for population size.

**Figure 1:**
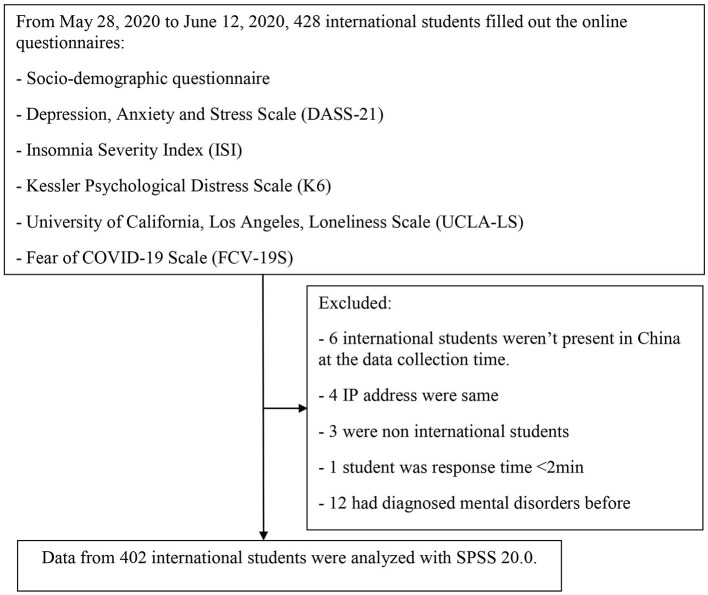
Flowchart of the study.

By substituting the values into the formula, the given value is *n* = 383.86. Therefore, a minimum sample size of 384 international students needs to be included in the sample. A total of 428 international students filled out the online questionnaires for this study. The inclusion criteria were as follows: (1) 18 years or older and (2) international students staying in the epidemic areas of mainland China during the outbreak of COVID-19. Exclusion criteria were (1) <18 years old, (2) international students who were not living in mainland China throughout the pandemic period, and (3) diagnosed and treated for mental illness before the outbreak. Out of 428 questionnaires filled up by the international students, 402 (93.9%) valid data were obtained. The detailed flow chart of this study is given in Figure 1.

### Measurements

#### Demographic Information

Demographic data were self-reported by the participants, including gender and age. The world's geographical regions are divided into four regions (Asia, Africa, Europe, or America). The geographical regions of China have been divided into seven regions (eastern, northern, southwest, northeast, central, southern, or northwest region). Information was collected on students' religion, marital status, living conditions, living place, education level, and areas of study, namely, Arts and Humanities, Medicine, Engineer, Agricultural, Business Studies, Social Sciences and Law, and Language. In the last question, participants were asked about the stay period in China. They were given four options: <1, <2, 2–3, and >3 years.

#### Depression, Anxiety, and Stress Scales

The Depression Anxiety Stress Scale (DASS-21) (21) was measured using the depression, anxiety, and stress during the past week through 21 items. Each of the three DASS-21 scales consists of seven items, and each item uses a 4-point Likert scale ranging from 0 (Did not apply to me at all) to 3 (Applied to me very much or most of the time). The total score of full scale ranges from 0 to 63, while the scale score of each dimension ranges from 0 to 21. The cumulative score for each subscale is computed by summing the scores for the items and multiplying by 2. Example items include “I couldn't seem to experience any positive feeling at all” for depression, “I was aware of dryness of my mouth” for anxiety, and “I found it hard to wind down” for stress. The cutoff points for a case finding are 10 for depression, 8 for anxiety, and 15 for stress. The depression subscale consists of items 3, 5, 10, 13, 16, 17, and 21 with scores ranging from normal (0–9), mild (10–13), moderate (14–20), severe (21–27), to extremely severe (28+). The anxiety subscale consists of items 2, 4, 7, 9, 15, 19, and 20, with scores ranging from normal (0–7), mild (8–9), moderate (10–14), severe (15–19), to extremely severe (20+). Finally, the stress subscale consists of items 1, 6, 8, 11, 12, 14, and 18, with scores ranging from normal (0–14), mild (15–18), moderate (19–25), severe (26–33), to extremely severe (34+). The DASS-21 is a reliable, easy-to-use screening instrument and has been well-received globally. We used the English version of the DASS-21 scales validated by past research (21). The internal consistency (Cronbach's alpha) in this study for depression, anxiety, and stress was found to be 0.81, 0.84, and 0.80, respectively, indicating good reliability.

#### Insomnia Severity Index

The Insomnia Severity Index (ISI) is a seven-item self-report questionnaire widely used to evaluate the nature, severity, and impact of insomnia (22). The ISI investigates participants' difficulty in falling asleep, remaining asleep, early waking, the satisfaction derived from the sleep pattern, impairments emerging in day-to-day functioning, awareness of sleep-related impairments, and stress levels caused by sleep problems in the last 2 weeks. Items are rated on a 5-point Likert scale, ranging from 0 (no problem) to 4 (very severe problem). The total score of the seven-item ISI ranges from 0 to 28. The total score was categorized into four different groups: no clinically significant insomnia (0–7), subthreshold insomnia (8–14), moderate insomnia (15–21), and severe insomnia (22–28) (23). In this study, the English version of the ISI scale score of 8 or higher indicates probable insomnia symptoms (22, 24). The English version of the ISI has good reliability and validity in general and clinical populations (24). The internal consistency (Cronbach's alpha) in this study was found to be 0.92, which indicates excellent reliability.

#### Kessler Psychological Distress Scale

The Kessler Psychological Distress Scale (K6) is a shortened, six-item version of the K10. In this study, the Kessler psychological distress scale assessed the participants' psychological distress (25). It contains six questions that ask participants to rate how often they have felt nervous, hopeless, restless, or fidgety, so depressed that nothing could cheer them up, that everything was an effort and worthless during the last 30 days. Answers were scored on a 5-point scale ranging from 1 (None of the time) to 5 (All of the time) and summed to create a continuous total score ranging from 6 to 30. We used the English version of the K6 scale validated by past research (26). A value of 13 or higher on the K6 indicates high or severe psychological distress. Values between 8 and 12 indicate moderate psychological distress, and a value between 0 and 7 denotes no psychological distress (27). The internal consistency (Cronbach's alpha) was found as 0.90, indicating excellent reliability in this study.

#### University of California, Los Angeles, Loneliness Scale

Loneliness was measured using the three-item short form of the revised University of California, Los Angeles, Loneliness Scale (UCLA-LS) (28). Three items assessed the frequency that an individual had felt a lack of companionship, left out, or isolated from others over the last week. Answers were scored on a 3-point scale ranging from 1 (Hardly ever) to 3 (Often) and summed to create a continuous total score ranging from 3 to 9. We used the English version of the UCLA-LS scale validated by past research (28). Participants with a score of 6 or higher were categorized as experiencing a high level of loneliness (29). The score then collapsed into one of two categories: a score of 3–5 reflects a negative screening for loneliness, and a score of 6–9 reflects a positive screening for loneliness. The internal consistency (Cronbach's alpha) in this study was found as 0.72, which indicates acceptable reliability.

#### Fear of COVID-19 Scale

The Fear of COVID-19 Scale (FCV-19S) is a self-report questionnaire to assess the level of fear associated with COVID-19. It was reliable and valid in determining COVID-19 fear among the general population (30). It consists of seven items (e.g., I am most afraid of coronavirus-19, my hands become clammy when I think about coronavirus-19) with a 5-point Likert scale response from 1 (strongly disagree) to 5 (strongly agree), and its total score range is 7–35. The higher the score indicates, the greater the fear of coronavirus-19 (30). Because no official severity for fear of COVID-19 scale was available, we used a severity scale using percentiles of FCV-19S score as follows: mild (≤ 17), moderate (18–23), and severe (≥24) (31). The internal consistency of the FCV-19S in the present study was excellent (Cronbach's α = 0.92).

### Statistical Analysis

Data analysis was performed using SPSS statistical software version 20.0 (IBM Corporation, Armonk, NY, USA). The Kolmogorov–Smirnov-test and Shapiro–Wilk-test were used to assess the normal distribution of variables. The original scores of the five measuring instruments were non-normally distributed. For this reason, we expressed median values with interquartile ranges (IQRs). The ranked data derived from each level's counts for symptoms of depression, anxiety, stress, insomnia, psychological distress, loneliness, and fear were presented as numbers and percentages. The non-parametric Mann–Whitney *U-*test and Kruskal–Wallis-test were applied to compare the severity of each symptom between two or more groups. Spearman correlations were performed to determine the relationships between levels of depression, anxiety, stress, insomnia, psychological distress, loneliness, and fear symptoms. In this study, binary logistic regression analysis was used to identify potential risk factors for psychological outcomes symptoms. Relationships between risk factors and psychological outcomes were expressed as crude odds ratio (COR) for univariate analyses and adjusted odds ratio (AOR) for multivariate analyses. Both were 95% confidence intervals (CIs), and *p* < 0.05 were regarded as statistically significant.

## Results

### Demographic Characteristics

A total of 402 international students aged between 18 and 40 years old from seven regions of China completed the questionnaire, of whom 340 (84.6%) were male, and 62 (15.4%) were female. One hundred sixty-two participants aged between 26 and 30 years old (40.3%). The majority of participants was from Asian countries (89.8%). More than half of the participants were from eastern regions (55.5%). Most of the participants belonged to Islam (78.1%) and were unmarried (72.9%). More than half of the participants lived with a roommate (52.2%), and their living place was a dormitory (79.6%). Lower than half of the participants had a bachelor's educational level (39.8%) and Engineering students (35.3%). One-third of the participants stayed in China for more than 3 years (35.3%). The median (IQR) scores on the depression, anxiety, stress, insomnia, psychological distress, loneliness, and fear symptoms for all participants were sufficient respectively in 18.0 (8.0–26.0), 18.0 (8.0–26.0), 16.0 (10.0–26.0), 13.0 (8.0–21.0), 10.0 (12.0–22.0), 2.0 (5.0–7.0), and 12.0 (17.0–29.0) (Table 1).

**Table 1:** Sample characteristics (*N* = 402).

**Factors**	**Participants, No. (%)**
Overall	402 (100.0)
**Gender**	
Male	340 (84.6)
Female	62 (15.4)
**Age (years)**	
18–25	129 (32.1)
26–30	162 (40.3)
31–35	85 (21.1)
36–40	26 (6.5)
**Geographical regions, World**	
Asia	361 (89.8)
Africa	28 (7.0)
Europe	8 (2.0)
America	5 (1.2)
**Geographical regions, China**	
Eastern	223 (55.5)
Northern	28 (7.0)
Northwest	21 (5.2)
Northeast	19 (4.7)
Central	52 (12.9)
Southern	14 (3.5)
Southwest	45 (11.2)
**Religion**	
Islam	314 (78.1)
Hinduism	20 (5.0)
Buddhist	33 (8.2)
Christian	30 (7.5)
Others	1 (0.2)
No religion	4 (1.0)
**Marital status**	
Unmarried	293 (72.9)
Married	108 (26.9)
Divorced/separated/widowed	1 (0.2)
**Living conditions**	
Alone	106 (26.4)
Roommate	210 (52.2)
Family	85 (21.1)
Other	1 (0.2)
**Living place**	
Dormitory	320 (79.6)
Hotel	11 (2.7)
Outside of the campus	71 (17.7)
**Education level**	
Bachelor	160 (39.8)
Master	112 (27.9)
Doctor/Ph.D.	123 (30.6)
Other	7 (1.7)
**Area of study**	
Arts and Humanities	14 (3.5)
Medicine	41 (10.2)
Engineer	142 (35.3)
Agricultural	56 (13.9)
Business studies	38 (9.5)
Social Sciences and Law	35 (8.7)
Language	57 (14.2)
Other	19 (4.7)
**Stay period in China (years)**	
<1	35 (8.7)
<2	132 (32.8)
2–3	93 (23.1)
>3	142 (35.3)
**Total score, median (IQR)**	
Depression symptoms	18.0 (8.0–26.0)
Anxiety symptoms	18.0 (8.0–26.0)
Stress symptoms	16.0 (10.0–26.0)
Insomnia symptoms	13.0 (8.0–21.0)
Psychological distress symptoms	10.0 (12.0–22.0)
Loneliness symptoms	2.0 (5.0–7.0)
Fear symptoms	12.0 (17–29.0)

*IQR, interquartile range*.

### The Severity of Psychological Outcomes and Associated Factors

**Figure 2:**
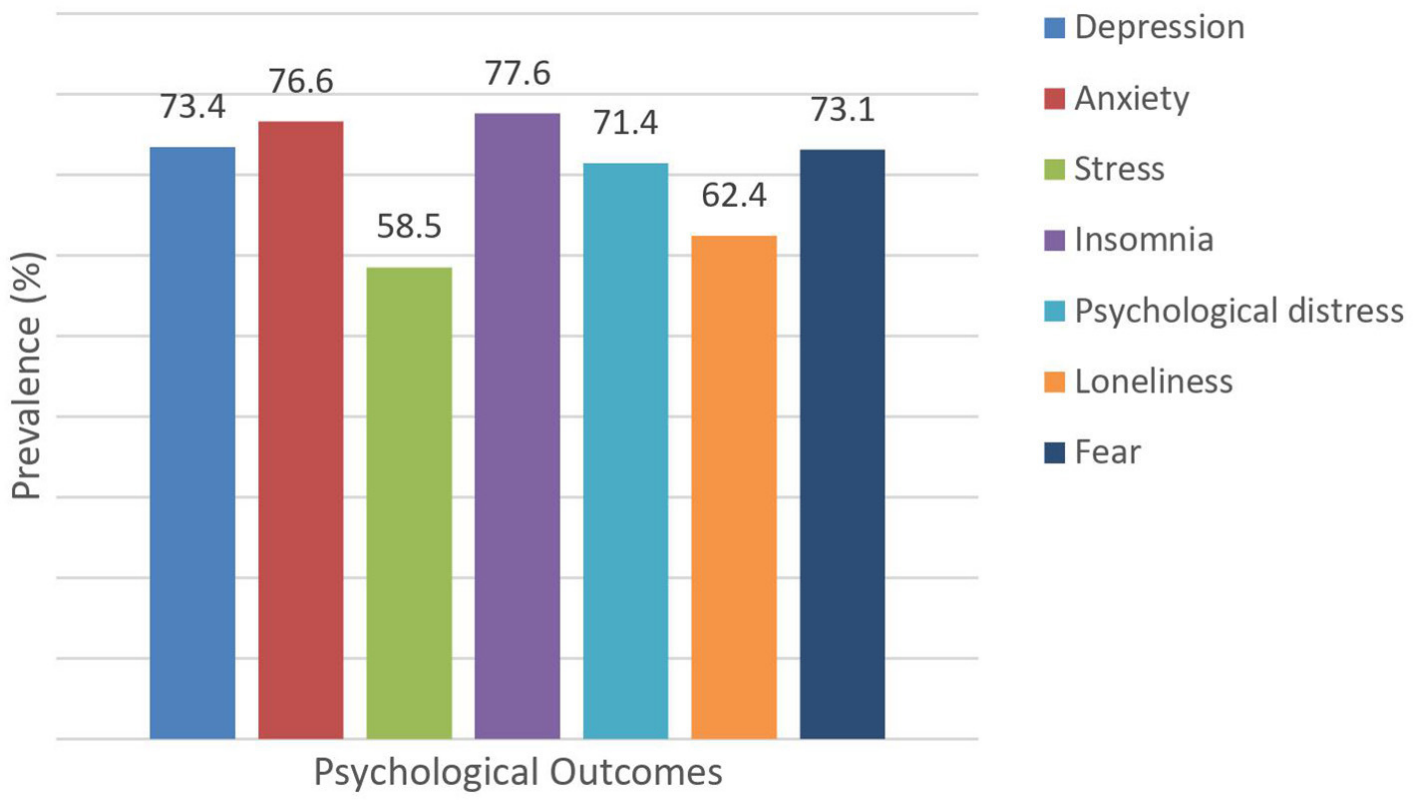
Prevalence of psychological outcomes during the COVID-19 pandemic.

A considerable proportion of participants had symptoms of depression (73.4%), anxiety (76.6%), stress (58.5%), insomnia (77.6%), psychological distress (71.4%), loneliness (62.4%), and fear (73.1%) (Figure 2). Male participants reported experiencing moderate to extremely severe symptoms of depression, anxiety, insomnia, and fear than the female participants. The prevalence of moderate to extremely severe symptoms of all the psychological outcomes was significantly higher in the age groups of 26–30 years old than in other age groups. Significantly moderate to extremely severe symptom levels of all the psychological outcomes were higher in central region students, except fear symptoms, than in other regions. Compared with those living alone and other, participants living with a roommate were associated with moderate to extremely severe symptoms of all the psychological outcomes. Participants who lived in the dormitory were significantly associated with moderate to extremely severe symptoms of depression, anxiety, stress, and insomnia. Bachelor students reported experiencing more severe symptoms of all the psychological outcomes except insomnia and fear. On the other hand, master students were significantly associated with more severe symptoms of insomnia and fear. Participants who were Arts and Humanities students were significantly associated with moderate to extremely severe symptoms of depression and anxiety, while Social Sciences and Law students were higher in insomnia and fear symptoms. On the other hand, Language students were significantly associated with more severe symptoms of psychological distress and loneliness. The prevalence of moderate to extremely severe symptoms of all the psychological outcomes was significantly higher in participants staying in China for <2 years compared with staying in China <1, 2–3, and >3 years (Table 2).

Table 2Severity categories of depression, anxiety, stress, insomnia, psychological distress, loneliness, and fear measurements in total cohort and subgroups.

**Table:** 

		**Gender**			**Age (years)**					**Geographical regions, World**					**Geographical regions, China**								**Religion**						
		**No. (%)**			**No. (%)**					**No. (%)**					**No. (%)**								**No. (%)**						
**Severity category**	**Total, No. (%)**	**Male**	**Female**	***P-*value**	**18–25**	**26–30**	**31–35**	**36–40**	***P* value**	**Asia**	**Africa**	**Europe**	**America**	***P-*value**	**Eastern**	**Northern**	**Northwest**	**Northeast**	**Central**	**Southern**	**Southwest**	***P-*value**	**Islam**	**Hinduism**	**Buddhist**	**Christian**	**Others**	**No religion**	***P-*value**
**Depression symptoms**																													
Normal to mild	153 (38.1)	122 (35.9)	31 (50.0)	0.03	49 (38.0)	47 (29.0)	37 (43.5)	20 (76.9)	0.00	133 (36.8)	18 (64.3)	1 (12.5)	1 (20.0)	0.01	113 (50.7)	9 (32.1)	3 (14.3)	6 (31.6)	7 (13.5)	6 (42.9)	9 (20.0)	0.00	121 (38.5)	12 (60.0)	8 (24.2)	8 (26.7)	1 (100.0)	3 (75.0)	0.03
Moderate to extremely severe	249 (61.9)	218 (64.1)	31 (50.0)		80 (62.0)	115 (71.0)	48 (56.5)	6 (23.1)		228 (63.2)	10 (35.7)	7 (87.5)	4 (80.0)		110 (49.3)	19 (67.9)	18 (85.7)	13 (68.4)	45 (86.5)	8 (57.1)	36 (80.0)		193 (61.5)	8 (40.0)	25 (75.8)	22 (73.3)	-	1 (25.0)	
**Anxiety symptoms**																													
Normal to mild	127 (31.6)	100 (29.4)	27 (43.5)	0.02	45 (34.9)	36 (22.2)	29 (34.1)	17 (65.4)	0.00	115 (31.9)	11 (39.3)	1 (12.5)	-	0.21	97 (43.5)	7 (25.0)	3 (14.3)	3 (15.8)	4 (7.7)	5 (35.7)	8 (17.8)	0.00	101 (32.2)	6 (30.0)	8 (24.2)	7 (23.3)	1 (100.0)	1 (100.0)	0.02
Moderate to extremely severe	275 (68.4)	240 (70.6)	35 (56.5)		84 (65.1)	126 (77.8)	56 (65.9)	9 (34.6)		246 (68.1)	17 (60.7)	7 (87.5)	5 (100.0)		126 (56.5)	21 (75.0)	18 (85.7)	16 (84.2)	48 (92.3)	9 (64.3)	37 (82.2)		213 (67.8)	14 (70.0)	25 (75.8)	23 (76.7)	-	-	
**Stress symptoms**																													
Normal to mild	210 (52.2)	171 (50.3)	39 (62.9)	0.06	68 (52.7)	75 (46.3)	45 (52.9)	22 (84.6)	0.00	186 (51.5)	17 (60.7)	5 (62.5)	2 (40.0)	0.67	145 (65.0)	15 (53.6)	7 (33.3)	8 (42.1)	10 (19.2)	6 (42.9)	19 (42.2)	0.00	165 (52.5)	11 (55.0)	15 (45.5)	14 (46.7)	1 (100.0)	4 (100.0)	0.34
Moderate to extremely severe	192 (47.8)	169 (49.7)	23 (37.1)		61 (47.3)	87 (53.7)	40 (47.1)	4 (15.4)		175 (48.5)	11 (39.3)	3 (37.5)	3 (60.0)		78 (35.0)	13 (46.4)	14 (66.7)	11 (57.9)	42 (80.8)	8 (57.1)	26 (57.8)		149 (47.5)	9 (45.0)	18 (54.5)	16 (53.3)	-	-	
**Insomnia symptoms**																													
No clinically significant to subthreshold	159 (39.6)	126 (37.1)	33 (53.2)	0.01	51 (39.5)	48 (29.6)	38 (44.7)	22 (84.6)	0.00	134 (37.1)	20 (71.4)	3 (37.5)	2 (40.0)	0.00	118 (52.9)	12 (42.9)	3 (14.3)	5 (26.3)	8 (15.4)	5 (35.7)	8 (17.8)	0.00	125 (39.8)	11 (55.0)	9 (27.3)	11 (36.7)	-	3 (75.0)	0.22
Moderate to severe	243 (60.4)	214 (62.9)	29 (46.8)		78 (60.5)	114 (70.4)	47 (55.3)	4 (15.4)		227 (62.9)	8 (28.6)	5 (62.5)	3 (60.0)		105 (47.1)	16 (57.1)	18 (85.7)	14 (73.7)	44 (84.6)	9 (64.3)	37 (82.2)		189 (60.2)	9 (45.0)	24 (72.7)	19 (63.3)	1 (100.0)	1 (25.0)	
**Psychological distress symptoms**																													
None	40 (10.0)	34 (10.0)	6 (9.7)	0.93	11 (8.5)	11 (6.8)	8 (9.4)	10 (38.5)	0.00	32 (8.9)	6 (21.4)	1 (12.5)	1 (20.0)	0.15	33 (14.8)	2 (7.1)	1 (4.8)	2 (10.5)	-	2 (14.3)	-	0.00	34 (10.8)	2 (10.0)	1 (3.0)	3 (10.0)	-	-	0.76
Moderate to severe	362 (90.0)	306 (90.0)	56 (90.3)		118 (91.5)	151 (93.2)	77 (90.6)	16 (61.5)		329 (91.1)	22 (78.6)	7 (87.5)	4 (80.0)		190 (85.2)	26 (92.9)	20 (95.2)	17 (89.5)	52 (100.0)	12 (85.7)	45 (100.0)		280 (89.2)	18 (90.0)	32 (97.0)	27 (90.0)	1 (100.0)	4 (100.0)	
**Loneliness symptoms**																													
Low	151 (37.6)	123 (36.2)	28 (45.2)	0.18	46 (35.7)	52 (32.1)	36 (42.4)	17 (65.4)	0.00	129 (35.7)	15 (53.6)	3 (37.5)	4 (80.0)	0.06	102 (45.7)	10 (35.7)	10 (47.6)	5 (26.3)	6 (11.5)	3 (21.4)	15 (33.3)	0.00	126 (40.1)	3 (15.0)	8 (24.2)	11 (36.7)	-	3 (75.0)	0.05
High	251 (62.4)	217 (63.8)	34 (54.8)		83 (64.3)	110 (67.9)	49 (57.6)	9 (34.6)		232 (64.3)	13 (46.4)	5 (62.5)	1 (20.0)		121 (54.3)	18 (64.3)	11 (52.4)	14 (73.7)	46 (88.5)	11 (78.6)	30 (66.7)		188 (59.9)	17 (85.0)	25 (75.8)	19 (63.3)	1 (100.0)	1 (25.0)	
**Fear symptoms**																													
Mild	108 (26.9)	83 (24.4)	25 (40.3)	0.00	41 (31.8)	32 (19.8)	24 (28.2)	11 (42.3)	0.03	91 (25.2)	12 (42.9)	3 (37.5)	2 (40.0)	0.16	86 (38.6)	4 (14.3)	3 (14.3)	3 (15.8)	7 (13.5)	1 (7.1)	4 (8.9)	0.00	82 (26.1)	7 (35.0)	8 (24.2)	7 (23.3)	1 (100.0)	3 (75.0)	0.13
Moderate to severe	294 (73.1)	257 (75.6)	37 (59.7)		88 (68.2)	130 (80.2)	61 (71.8)	15 (57.7)		270 (74.8)	16 (57.1)	5 (62.5)	3 (60.0)		137 (61.4)	24 (85.7)	18 (85.7)	16 (84.2)	45 (86.5)	13 (92.9)	41 (91.1)		232 (73.9)	13 (65.0)	25 (75.8)	23 (76.7)	-	1 (25.0)

**Table:** 

		**Marital status**				**Living conditions**					**Living place**				**Education level**					**Area of study**									**Stay period in China (years)**				
		**No. (%)**				**No. (%)**					**No. (%)**				**No. (%)**					**No. (%)**									**No. (%)**				
**Severity category**	**Total, No. (%)**	**Unmarried**	**Married**	**Div/Sep/Wid**	***P-*** **value**	**Alone**	**Roommate**	**Family**	**Other**	***P-*** **value**	**Dormitory**	**Hotel**	**Outside of the campus**	***P-*** **value**	**Bachelor**	**Master**	**Doctor/Ph.D**.	**Other**	***P-*** **value**	**Arts and Humanities**	**Medicine**	**Engineer**	**Agricultural**	**Business studies**	**Social Sciences and Law**	**Language**	**Other**	***P*** **value**	** <1**	** <2**	**2–3**	**>3**	***P-*** **value**
**Depression symptoms**																																	
Normal to mild	153 (38.1)	120 (41.0)	33 (30.6)	-	0.12	76 (71.7)	37 (17.6)	39 (45.9)	1 (100.0)	0.00	105 (32.8)	9 (81.8)	39 (54.9)	0.00	46 (28.8)	35 (31.3)	66 (53.7)	6 (85.7)	0.00	3 (21.4)	21 (51.2)	40 (28.2)	27 (48.2)	13 (34.2)	9 (25.7)	24 (42.1)	16 (84.2)	0.00	23 (65.7)	21 (15.9)	30 (32.3)	79 (55.6)	0.00
Moderate to extremelysevere	249 (61.9)	173 (59.0)	75 (69.4)	1 (100.0)		30 (28.3)	173 (82.4)	46 (54.1)	-		215 (67.2)	2 (18.2)	32 (45.1)		114 (71.3)	77 (68.8)	57 (46.3)	1 (14.3)		11 (78.6)	20 (48.8)	102 (71.8)	29 (51.8)	25 (65.8)	26 (74.3)	33 (57.9)	3 (15.8)		12 (34.3)	111 (84.1)	63 (67.7)	63 (44.4)	
**Anxiety symptoms**																																	
Normal to mild	127 (31.6)	95 (32.4)	32 (29.6)	-	0.68	62 (58.5)	31 (14.8)	33 (38.8)	1 (100.0)	0.00	90 (28.1)	6 (54.5)	31 (43.7)	0.01	39 (24.4)	32 (28.6)	53 (43.1)	3 (42.9)	0.00	3 (21.4)	17 (41.5)	37 (26.1)	21 (37.5)	9 (23.7)	8 (22.9)	16 (28.1)	16 (84.2)	0.00	21 (60.0)	17 (12.9)	21 (22.6)	68 (47.9)	0.00
Moderate to extremely severe	275 (68.4)	198 (67.6)	76 (70.4)	1 (100.0)		44 (41.5)	179 (85.2)	52 (61.2)	-		230 (71.9)	5 (45.5)	40 (56.3)		121 (75.6)	80 (71.4)	70 (56.9)	4 (57.1)		11 (78.6)	24 (58.5)	105 (73.9)	35 (62.5)	29 (76.3)	27 (77.1)	41 (71.9)	3 (15.8)		14 (40.0)	115 (87.1)	72 (77.4)	74 (52.1)	
**Stress symptoms**																																	
Normal to mild	210 (52.2)	165 (56.3)	45 (41.7)	-	0.02	93 (87.7)	70 (33.3)	46 (54.1)	1 (100.0)	0.00	155 (48.4)	9 (81.8)	46 (64.8)	0.00	70 (43.8)	56 (50.0)	78 (63.4)	6 (85.7)	0.00	7 (50.0)	28 (68.3)	57 (40.1)	30 (53.6)	20 (52.6)	15 (42.9)	36 (63.2)	17 (89.5)	0.00	29 (82.9)	44 (33.3)	38 (40.9)	99 (69.7)	0.00
Moderate to extremely severe	192 (47.8)	128 (43.7)	63 (58.3)	1 (100.0)		13 (12.3)	140 (66.7)	39 (45.9)	-		165 (51.6)	2 (18.2)	25 (35.2)		90 (56.3)	56 (50.0)	45 (36.6)	1 (14.3)		7 (50.0)	13 (31.7)	85 (59.9)	26 (46.4)	18 (47.4)	20 (57.1)	21 (36.8)	2 (10.5)		6 (17.1)	88 (66.7)	55 (59.1)	43 (30.3)	
**Insomnia symptoms**																																	
No clinically significant to subthreshold	159 (39.6)	122 (41.6)	37 (34.3)	-	0.29	80 (75.5)	36 (17.1)	42 (49.4)	1 (100.0)	0.00	113 (35.3)	8 (72.7)	38 (53.5)	<0.001	51 (31.9)	33 (29.5)	69 (56.1)	6 (85.7)	0.00	4 (28.6)	21 (51.2)	38 (26.8)	30 (53.6)	15 (39.5)	9 (25.7)	26 (45.6)	16 (84.2)	0.00	21 (60.0)	23 (17.4)	28 (30.1)	87 (61.3)	0.00
Moderate to severe	243 (60.4)	171 (58.4)	71 (65.7)	1 (100.0)		26 (24.5)	174 (82.9)	43 (50.6)	-		207 (64.7)	3 (27.3)	33 (46.5)		109 (68.1)	79 (70.5)	54 (43.9)	1 (14.3)		10 (71.4)	20 (48.8)	104 (73.2)	26 (46.4)	23 (60.5)	26 (74.3)	31 (54.4)	3 (15.8)		14 (40.0)	109 (82.6)	65 (69.9)	55 (38.7)	
**Psychological distress symptoms**																																	
None	40 (10.0)	30 (10.2)	10 (9.3)	-	0.90	17 (16.0)	8 (3.8)	15 (17.6)	-	0.00	29 (9.1)	-	11 (15.5)	0.14	7 (4.4)	6 (5.4)	23 (18.7)	4 (57.1)	0.00	2 (14.3)	6 (14.6)	10 (7.0)	14 (25.0)	3 (7.9)	2 (5.7)	2 (3.5)	1 (5.3)	0.00	6 (17.1)	7 (5.3)	6 (6.5)	21 (14.8)	0.01
Moderate to severe	362 (90.0)	263 (89.8)	98 (90.7)	1 (100.0)		89 (84.0)	202 (96.2)	70 (82.4)	1 (100.0)		291 (90.9)	11 (100.0)	60 (84.5)		153 (95.6)	106 (94.6)	100 (81.3)	3 (42.9)		12 (85.7)	35 (85.4)	132 (93.0)	42 (75.0)	35 (92.1)	33 (94.3)	55 (96.5)	18 (94.7)		29 (82.9)	125 (94.7)	87 (93.5)	121 (85.2)	
**Loneliness symptoms**																																	
Low	151 (37.6)	95 (32.4)	56 (51.9)	-	0.31	51 (48.1)	55 (26.2)	44 (51.8)	1 (100.0)	0.00	113 (35.3)	2 (18.2)	36 (50.7)	0.02	54 (33.7)	36 (32.1)	58 (47.2)	3 (42.9)	0.06	4 (28.6)	23 (56.1)	40 (28.2)	31 (55.4)	16 (42.1)	11 (31.4)	14 (24.6)	12 (63.2)	0.00	21 (60.0)	30 (22.7)	36 (38.7)	64 (45.1)	0.00
High	251 (62.4)	198 (67.6)	52 (48.1)	1 (100.0)		55 (51.9)	155 (73.8)	41 (48.2)	-		207 (64.7)	9 (81.8)	35 (49.3)		106 (66.3)	76 (67.9)	65 (52.8)	4 (57.1)		10 (71.4)	18 (43.9)	102 (71.8)	25 (44.6)	22 (57.9)	24 (68.6)	43 (75.4)	7 (36.8)		14 (40.0)	102 (77.3)	57 (61.3)	78 (54.9)	
**Fear symptoms**																																	
Mild	108 (26.9)	81 (27.6)	27 (25.0)	-	0.72	58 (54.7)	28 (13.3)	21 (24.7)	1 (100.0)	0.00	81 (25.3)	5 (45.5)	22 (31.0)	0.23	38 (23.8)	23 (20.5)	45 (36.6)	2 (28.6)	0.03	3 (21.4)	22 (53.7)	26 (18.3)	16 (28.6)	10 (26.3)	4 (11.4)	15 (26.3)	12 (63.2)	0.00	14 (40.0)	15 (11.4)	18 (19.4)	61 (43.0)	0.00
Moderate to severe	294 (73.1)	212 (72.4)	81 (75.0)	1 (100.0)		48 (45.3)	182 (86.7)	64 (75.3)	-		239 (74.7)	6 (54.5)	49 (69.0)		122 (76.3)	89 (79.5)	78 (63.4)	5 (71.4)		11 (78.6)	19 (46.3)	116 (81.7)	40 (71.4)	28 (73.7)	31 (88.6)	42 (73.7)	7 (36.8)		21 (60.0)	117 (88.6)	75 (80.6)	81 (57.0)	

### Correlations of Psychological Outcomes

Table 3 presents Spearman's correlation of all study variables. The results indicated that depression, anxiety, stress, insomnia, psychological distress, loneliness, and fear symptoms significantly and positively correlated with one another (*p* < 0.01). It was rare that five scales were significantly and positively correlated with one another compared with other research.

**Table 3:** Spearman's correlations of psychological outcomes.

**Scales**	**1**	**2**	**3**	**4**	**5**	**6**	**7**
1	1						
2	0.794^**^	1					
3	0.796^**^	0.790^**^	1				
4	0.699^**^	0.704^**^	0.704^**^	1			
5	0.767^**^	0.738^**^	0.769^**^	0.795^**^	1		
6	0.522^**^	0.527^**^	0.538^**^	0.591^**^	0.593^**^	1	
7	0.691^**^	0.704^**^	0.641^**^	0.674^**^	0.688^**^	0.535^**^	1

** *p < 0.01*.

*1, Depression; 2, anxiety; 3, stress; 4, insomnia; 5, psychological distress; 6, loneliness; 7, fear*.

### Risk Factors of Psychological Outcomes

We performed binary logistic regression analyses to identify demographic and relevant contextual factors associated with psychological outcomes. The univariate logistic regression analyses (Supplementary Table 1) showed that male participants presented higher depression, anxiety, insomnia, and fear symptoms than female students. A significance of all the psychological outcomes was found among 26–30-year-old students rather than in other age groups. Depression, anxiety, stress, insomnia, and fear symptoms were more common among eastern region students compared to other regions. Compared with those living in a hotel and outside, participants who lived in the dormitory were more likely to report all the psychological outcomes except psychological distress and fear symptoms. Depression, insomnia, and psychological distress were more common among bachelor students than in other education levels. Compared with other areas of study, Arts and Humanities, Engineering, Social Sciences and Law, and Language students were significantly associated with the symptoms of depression, anxiety, stress, insomnia, loneliness, and fear. Students whose staying period in China was <2 years were significantly associated with all the psychological outcomes than other students.

The multivariate logistic regression analysis showed that 31–35-year-old students were more likely to have depression, anxiety, stress, insomnia, and psychological distress symptoms than other age groups. Compared with other areas of study, participants of Engineering, Business, Social Sciences and Law, and Language students were significantly associated with the symptoms of depression, anxiety, insomnia, and fear. Compared to those whose stayed period in China 3 years or more, students whose staying period in China <1 year were associated with depression and loneliness symptoms. On the other hand, those who were in China for <2 years had all kinds of psychological outcomes except psychological distress and loneliness symptoms. The detailed results of multivariate logistic regression analysis are shown in Supplementary Table 2.

## Discussion

The first broad range study investigates the magnitude of psychological outcomes and associated factors among international students currently living in China during the COVID-19 pandemic. This cross-sectional survey enrolled 402 respondents and revealed a high prevalence of psychological effects among international students during the COVID-19 epidemic residing in China. Overall, more than half of all participants reported depression, anxiety, stress, insomnia, psychological distress, loneliness, and fear symptoms. This high prevalence of mental health symptoms is supported and consistent with previous studies in various age groups, gender, marital status, education, place of living, fields, and different countries.

The present study found that 73.4, 76.6, and 58.5% of the participants had depression, anxiety, and stress symptoms. This study's rates were lower than the previous studies. For example, a web-based cross-sectional survey of 476 university students living in Bangladesh utilizing the Patient Health Questionnaire (PHQ-9) and Generalized Anxiety Disorder (GAD-7) found that 82.4% of students have mild to severe depressive symptoms, and 87.7% of students have mild to severe anxiety symptoms (32). In Jordan, an online survey conducted in April 2020 involved 456 undergraduate students utilizing the DASS-21 who reported that the majority of students had symptoms of depression (74.1%), anxiety (59.6%), and stress (61.2%) (33). Another study that involved 2,086 college students regarding the impact of COVID-19 on their mental health in April 2020 found that 91% of the participants had anxiety or stress symptoms (34).

Our results showed that the prevalence of insomnia symptoms was 77.6%, which was greater than that in previous studies. A recent systematic scoping review of 78 articles related to various professions like university students found that the prevalence of sleeping disorders ranged from 2.3 to 76.6% (35). Our study found that 71.4% of the participants reported psychological distress symptoms. These rates were higher than in the previous studies. For example, a longitudinal study of 622 nursing students in Italy, utilizing the GHQ-12, found that >70% had significant levels of psychological distress (36). A previous study investigating predictive factors for impaired mental health to the COVID-19 pandemic in 2020 among 549 medical students using PHQ-9, GAD-7, ISI, and K6 scales in Morocco indicated that 62.3, 74.6, 62.6, and 69% reported anxiety, depression, insomnia, and distress symptoms, respectively (37). However, Zhang et al. (38) revealed that the detection rate of anxiety symptoms was about 15% in medical students from Mongolia medical colleges in mainland China, and 77% of the students had shown distress symptoms in the past 7 days.

Our findings showed that the prevalence of loneliness symptoms was 62.4%, lower than the other studies (39, 40). In a prospective cohort study of 213 Art students in the Netherlands, utilizing the loneliness scale, researchers found that at least 75% of the participants dealt with moderate to very severe loneliness in all 3 months during the COVID-19 lockdown (40). Furthermore, the results indicated that the prevalence of fear symptoms was 73.1%, higher than in the earlier studies (41). A survey conducted in 912 nursing students and graduates during the last 18 months from public and private universities of Mexico used the fear of COVID-19 scale to find fear regarding COVID-19 in 50.3% (41).

In this study, the findings revealed that males were more likely to have depressive, anxiety, insomnia, and fear symptoms than female students. A recent online cross-sectional survey performed in Delhi NCR, Maharashtra, and Tamil Nadu during May 2020 investigated 335 dental students and practitioners who used PHQ-9 scores found that those who were depressed were likely to be male than female (42). However, the result of this study was consistent with the other research conducted in China that male students were significantly more anxious than female students (43). Furthermore, another study found that male students had a higher rate of insomnia than female students (27.7 vs. 20.0%) (44). However, in our research, we found that male sex was associated with fear symptoms during the COVID-19 outbreak, which differed from the previous studies, indicating that female students showed higher levels of fear of COVID-19 than male students (45). It could be the reason that a male student was more likely to engage in risk-taking behaviors (46). In addition, the majority of the students in this study were male (84.6%).

Our study demonstrated that participants aged 26–30 years reported statistically significantly associated psychological outcomes. The participants of this group were highly pressurized in multifetch levels that impacted heavily on their mental health. This finding might cause more anxiousness about the study, career, family, and sometimes financial management of young participants. Many studies found that young students were at higher risks of general psychiatric disorders, stress, anxiety, depression, loneliness, psychological distress, suicidal ideation, insomnia, and post-traumatic stress symptoms (46–49). A recent cross-sectional survey performed in India during May 2020 investigated 335 dental students and practitioners who used PHQ-9 scores and found that those who were depressed were likely to be younger than 30 years old (42).

The present study also demonstrated that central region students reported significant association with all the psychological outcomes except fear symptoms. There are eight provinces in central regions, and Hubei is one of them. Wuhan is the capital of Hubei Province in the People's Republic of China. In December 2019, the outbreak of COVID-19 began in Wuhan. This city first implemented a Level 1 response to the public health emergency and a lockdown on January 23, 2020, due to the high fatality rate (50). During the lockdown period, students did not get permission to go outside the campus. Most of the people, except for those involved in epidemic prevention and control, the police, and few workers of necessary industries, were required to stay at home (51, 52). Under the government policies on COVID-19, universities of China, especially in the Wuhan region, issued strict rules for local and international students to prevent the transmission of the virus in the university community. This situation has created a panic situation among the students, especially those living in the earthquake's epicenter.

After Wuhan city, the government of all provinces in China implemented a Level 1 response to the public health emergency on January 29, 2020 (53). Earlier studies have shown that public health emergencies have a significant impact on the mental health of college students (54). Hence, all universities in China were mandated to be closed in the spring of 2020. A previous study investigating the psychological impact of the COVID-19 outbreak in 2020 developed by using a questionnaire among 504 valid responses from international students in Hubei province, China, found that it was 2.12 times greater in students from Wuhan than in those from other areas (55). It may be because the respondents in affected areas paid more attention to the safety of their families (56). However, another study of 2,485 students from six universities investigated using online survey versions of the PCL-C and PHQ-9 found that those living in the worst-hit areas were at the highest risk of developing post-traumatic stress disorder (PTSD) and depression (13).

Our study revealed that participants who lived with a roommate were more likely to report moderate to extremely severe symptoms of all the psychological outcomes. In a recent study from the USA, students living with roommates showed secondary associations between physiologically and environmentally related sleep hygiene practices and depressive symptoms (57). A cross-sectional survey among final-year dental undergraduate students in a dental teaching institution in Bangalore, India, found that students who had been staying with roommates were least commonly reported to have mental health problems (58). Since bachelor and master's students living with a roommate in a dormitory, so they had more talk to each other about COVID-19 rather than other topics during the pandemic. Sometimes, they got insufficient information or got misinformation (“fake news”). A recent study found that inadequate details (59) or misinformation on COVID-19 (60) was associated with poorer mental health (61).

Our findings indicated that participants whose living places were dormitories were significantly associated with psychological outcomes except for psychological distress and fear symptoms. A recent study conducted in the United Arab Emirates on 433 students has found that students staying in a shared house or dorm (hostel) are more anxious about COVID-19 than those staying in a villa or apartment (62). They were cut off from meeting others except for virtual meetings. It posed heavy tension and other mental disorders among them due to a lack of communication. It might be that the concerned authorities strictly monitored students who lived in dormitories during the pandemic. However, they were allowed to go outside for a limited time purchasing daily commodities. It created an adverse effect on their minds, followed by mental health problems. For students living with dorms or shared houses, other factors such as online classes and exams, financial crisis, and null social gatherings adversely affected their minds. It is probably the first time that international students took part in online courses and exams. Since it was a new teaching and learning idea, many did not get used to it, consequently creating anxiousness.

The current study found that bachelor students reported significant association with depression, anxiety, stress, and psychological distress symptoms, consistent with previous studies (63, 64). International students who are staying far from their parents/loved ones are at a higher risk of developing mental problems such as anxiety and depression (65). They are worried about their health and education and have a massive concern for the well-being of their families (65, 66). A previous study investigating the psychological impact of the COVID-19 outbreak in 2020 developed by using a questionnaire among 504 valid responses from international students in Hubei province, China, found that the bachelor and Ph.D. students were more likely to be affected than the master students (55). Long-term living students had better knowledge about the illness, more adaptability, adjustment power, and prevention measures than the freshers, which could further shelter them from mental health symptoms. Due to long periods of staying, they are mostly well-acquainted with the local people, culture, customs, food habits, and environment, which created a plus point for them to tackle the worst situation in the pandemic. Newcomers, on the contrary, were devoid of assimilation process of the local culture and customs that led them to pose stress and anxiety during the pandemic.

This present study revealed that Arts and Humanities, Engineering, Social Sciences and Law, and Language students reported significant association with all the psychological outcomes except psychological distress symptoms, consistent with the previous studies (43, 46, 47, 67, 68). Odriozola-González et al. conducted a study of 2,530 participants at the University of Valladolid in Spain at the beginning of the COVID-19 outbreak. Their study found that participants who studied arts and humanities, social sciences, and law had higher scores of anxiety, depression, stress, and impact of the event than those who studied other subjects. A recent study of the psychological impact of the COVID-19 outbreak on 3,936 students in France found that those who studied in a language program had significantly more anxiety symptoms than those in other programs (69). However, results from another study in the 362 different medical and engineering colleges of Karachi from 2018, evaluated using the HAM-D, showed that the rate of depression was higher in engineering students than in medical students (48). Moreover, several studies evidence that the health sciences or engineering area students were found to present higher symptomatology scores than those in others (70, 71).

Our study showed that participants staying in China for <2 years were significantly associated with all the psychological outcomes. Our findings were different from previous findings of a survey conducted during the pandemic in China. It noted that international students who had been in China for <3 years suffered 2.19 times more than the students who had been here for 1 or 2 years (55). It may be because the students with a more extended stay in China reported more concerns and consequences than the students who stayed for a short period. It may be associated with the respondent's age and marital status. However, another study found that international students staying in another country for more than 1 year were more depressed than local-born students (72). Future epidemiological studies should emphasize psychopathological variations and temporality of mental health problems in different populations. The mental health of international students is also essential. Nonetheless, multipronged interventions should be developed and adopted to address the existing psychosocial challenges and promote mental health amid the COVID-19 pandemic.

## Strengths and Limitations

The strength of this study includes its extensive geographic coverage from 7 regions of inter-26 provincial level of China, 84 different types of universities, 45 country's international students, and the critical study period. Besides, to the best of our knowledge, this is the first nationwide study that systematically investigated the mental health outcomes and associated factors by standardized rating scales among international students living in China during the COVID-19 pandemic. This study finding may provide helpful information for government leaders and higher education institutions to recognize high-risk international students and design a framework for acute and long-term psychological services for them. This finding may also help the government to focus more on international students' mental health while combating the COVID-19 pandemic. Our investigation output will significantly impact psychiatry and public mental health and is conducive to psychiatrists, clinicians, and investigators in their research and deliver valuable information for universities authorities, policymakers, healthcare providers, and government officials. Additionally, this study could help them develop better prevention and treatment plans for their patients, general people, and local and international students, and mental health promotion globally.

Like all other studies, this study also has several limitations. First, the study was relatively small. Second, most of the participants in the current survey were from male students and Asian countries, which might have skewed the results. Third, the self-administered instruments can predict with some level of assurance that a person will meet the full criteria for a psychological disorder. However, the instruments themselves do not serve to diagnose these disorders. They should not take the place of complete diagnostic evaluation by experts. Fourth, the study's cross-sectional design did not permit the elucidation of causal relationships. Finally, the results may only reflect the current mental health status during the epidemic. Longitudinal follow-up studies are needed to determine the possible long-term mental health consequences among international students during the COVID-19 pandemic.

## Conclusion

The present study is the first broad range study investigating the magnitude of psychological outcomes and associated factors by standardized rating scales among international students living in China during the COVID-19 pandemic. A higher prevalence of psychological symptoms was found among the international students living in China during COVID-19 and risk factors. This study implies that universities need to take measures to prevent, identify, and deal with the mental health problems of international students during the COVID-19 pandemic. The findings of this study provide a scientific foundation in mental health interventions or support and practical strategies aimed at reminding researchers, university authorities, healthcare providers, and government officials to take precautions.

## Data Availability Statement

The raw data supporting the conclusions of this article will be made available by the authors, without undue reservation.

## Ethics Statement

The studies involving human participants were reviewed and approved by Ethics Committee of the First Affiliated Hospital, Zhejiang University School of Medicine. The patients/participants provided their written informed consent to participate in this study.

## Author Contributions

AM and JL: conceptualization and methodology. AM: formal analysis and writing—original draft. AM and LN: data curation. AM, JL, LN, SH, and YX: writing—review and editing. All authors contributed to the article and approved the submitted version.

## Conflict of Interest

The authors declare that the research was conducted in the absence of any commercial or financial relationships that could be construed as a potential conflict of interest.

## Publisher's Note

All claims expressed in this article are solely those of the authors and do not necessarily represent those of their affiliated organizations, or those of the publisher, the editors and the reviewers. Any product that may be evaluated in this article, or claim that may be made by its manufacturer, is not guaranteed or endorsed by the publisher.
