# Supplementary material for: Psychological Outcomes and Associated Factors Among the International Students Living in China During the COVID-19 Pandemic
Source: Front Psychiatry. 2021 Aug 13;12:707342. doi: 10.3389/fpsyt.2021.707342 (PMC8414650; doi:10.3389/fpsyt.2021.707342)
Supplement: Supplementary file 1 [file Data_Sheet_1.pdf]

Supplementary Material

Supplement Table S1. Risk Factors for Psychological Outcomes Identified by Binary Logistic Regression Analysis (univariate analysis).

	Depression		Anxiety		Stress		Insomnia		Psychological Distress		Loneliness		Fear	
**Variables**	**COR (95% CI)**	***P* value**	**COR (95% CI)**	***P* value**	**COR (95% CI)**	***P* value**	**COR (95% CI)**	***P* value**	**COR (95% CI)**	***P* value**	**COR (95% CI)**	***P* value**	**COR (95% CI)**	***P* value**
**Gender**														
Male	1.78 (1.03-3.08)	.03	1.85 (1.06-3.22)	.02	1.67 (0.96-2.92)	.06	1.93 (1.12-3.33)	.01	0.96 (0.38-2.40)	.93	0.68 (0.39-1.18)	.18	1.46 (0.75-2.81)	.01
Female	1 [Reference]		1 [Reference]		1 [Reference]		1 [Reference]		1 [Reference]		1 [Reference]		1 [Reference]	
**Age (years)**														
18-25	5.44 (2.04-14.4)	.00	3.52 (1.45-8.54)	.00	4.93 (1.61-15.1)	.00	8.41 (2.73-25.8)	.00	6.70 (2.45-18.2)	.00	0.29 (0.12-0.71)	.00	1.57 (0.66-3.72)	.30
26-30	8.15 (3.08-21.5)	.00	6.61 (2.71-16.0)	.00	6.38 (2.10-19.3)	<.001	13.0 (4.27-39.9)	.00	8.58 (3.15-23.3)	.00	0.25 (0.10-0.59)	.00	2.97 (1.25-7.10)	.01
31-35	4.32 (1.57-11.8)	.00	3.64 (1.44-9.19)	.00	4.88 (1.55-15.3)	.00	6.80 (2.15-21.4)	<.001	6.01 (2.05-17.6)	<.001	0.38 (0.15-0.97)	.04	1.86 (0.75-4.63)	.18
36-40	1 [Reference]		1 [Reference]		1 [Reference]		1 [Reference]		1 [Reference]		1 [Reference]		1 [Reference]	
**Geographical regions, World**														
Asia	0.42 (0.04-3.87)	.45	NA	NA	0.62 (0.10-3.79)	.61	1.12 (0.18-6.84)	.89	2.57 (0.27-23.6)	.40	0.13 (0.01-1.25)	.07	1.97 (0.32-12.0)	.45
Africa	0.13 (0.01-1.41)	.09	NA	NA	0.43 (0.06-3.01)	.39	0.26 (0.03-1.90)	.18	0.91 (0.08-9.80)	.94	0.28 (0.02-2.91)	.29	0.88 (0.12-6.18)	.90
Europe	1.75 (0.08-36.2)	.71	NA	NA	0.40 (0.04-3.95)	.43	1.11 (0.11-10.9)	.92	1.75 (0.08-36.2)	.71	0.15 (0.01-2.05)	.15	1.11 (0.11-10.9)	.92
America	1 [Reference]		1 [Reference]		1 [Reference]		1 [Reference]		1 [Reference]		1 [Reference]		1 [Reference]	
**Geographical regions, China**														
Eastern	0.16 (0.04-0.56)	.00	0.21 (0.06-0.75)	.01	0.26 (0.10-0.69)	.00	0.14 (0.04-0.51)	.00	0.28 (0.03-2.21)	.23	0.92 (0.37-2.27)	.86	0.26 (0.07-0.92)	.03
Northern	0.35 (0.08-1.51)	.16	0.50 (0.11-2.22)	.36	0.43 (0.13-1.40)	.16	0.22 (0.05-0.93)	.04	0.65 (0.05-7.68)	.73	0.61 (0.19-1.93)	.40	1.00 (0.19-5.03)	1.0
Southwest	0.66 (0.16-2.76)	.57	0.77 (0.18-3.25)	.72	0.68 (0.23-2.02)	.49	0.77 (0.18-3.25)	.72	NA	NA	0.55 (0.19-1.58)	.26	1.70 (0.34-8.43)	.51
Northeast	0.36 (0.07-1.71)	.20	0.88 (0.15-5.04)	.89	0.68 (0.19-2.48)	.56	0.46 (0.09-2.29)	.34	0.42 (0.03-5.10)	.50	0.39 (0.10-1.48)	.16	0.88 (0.15-5.04)	.89
Central	1.07 (0.24-4.60)	.92	2.00 (0.40-9.82)	.39	2.10 (0.67-6.56)	.20	0.91 (0.21-3.85)	.90	NA	NA	0.14 (0.04-0.48)	.00	1.07 (0.24-4.60)	.92
Southern	0.22 (0.04-1.11)	.06	0.30 (0.05-1.54)	.15	0.66 (0.16-2.68)	.56	0.30 (0.05-1.54)	.15	0.30 (0.02-3.67)	.34	0.30 (0.06-1.39)	.12	2.16 (0.20-23.2)	.52
Northwest	1 [Reference]		1 [Reference]		1 [Reference]		1 [Reference]		1 [Reference]		1 [Reference]		1 [Reference]	

**Religion**														
Islam	4.78 (0.49-46.5)	.17	NA	NA	NA	NA	4.53 (0.46-44.1)	.19	NA	NA	0.22 (0.02-2.17)	.19	8.48 (0.87-82.7)	.06
Hinduism	2.00 (0.17-22.7)	.57	NA	NA	NA	NA	2.45 (0.21-27.8)	.46	NA	NA	0.05 (0.004-0.77)	.03	5.57 (0.48-64.0)	.16
Buddhist	9.37 (0.85-103.2)	.06	NA	NA	NA	NA	8.00 (0.73-87.2)	.08	NA	NA	0.10 (0.01-1.17)	.06	9.37 (0.85-103.2)	.06
Christian	8.25 (0.74-91.2)	.08	NA	NA	NA	NA	5.18 (0.47-56.0)	.17	NA	NA	0.19 (0.01-2.08)	.17	9.85 (0.88-110.4)	.06
Others	NA	NA	NA	NA	NA	NA	NA	NA	NA	NA	NA	NA	NA	NA
No religion	1 [Reference]		1 [Reference]		1 [Reference]		1 [Reference]		1 [Reference]		1 [Reference]		1 [Reference]	
**Marital status**														
Unmarried	NA	NA	NA	NA	NA	NA	NA	NA	NA	NA	NA	NA	NA	NA
Married	NA	NA	NA	NA	NA	NA	NA	NA	NA	NA	NA	NA	NA	NA
Divorced/Separated/ Widowed	1 [Reference]		1 [Reference]		1 [Reference]		1 [Reference]		1 [Reference]		1 [Reference]		1 [Reference]	
**Living conditions**														
Alone	NA	NA	NA	NA	NA	NA	NA	NA	NA	NA	NA	NA	NA	NA
Roommate	NA	NA	NA	NA	NA	NA	NA	NA	NA	NA	NA	NA	NA	NA
Family	NA	NA	NA	NA	NA	NA	NA	NA	NA	NA	NA	NA	NA	NA
Other	1 [Reference]		1 [Reference]		1 [Reference]		1 [Reference]		1 [Reference]		1 [Reference]		1 [Reference]	
**Living place**														
Dormitory	2.49 (1.48-4.20)	<.001	1.98 (1.16-3.36)	.01	1.95 (1.14-3.34)	.01	2.10 (1.25-3.54)	.00	1.84 (0.87-3.88)	.11	0.53 (0.31-0.89)	.01	1.32 (0.75-2.32)	.32
Hotel	0.27 (0.05-1.34)	.11	0.64 (0.18-2.31)	.50	0.40 (0.08-2.04)	.27	0.43 (0.10-1.76)	.24	NA	NA	0.21 (0.04-1.07)	.06	0.53 (0.14-1.95)	.34
Outside of the campus	1 [Reference]		1 [Reference]		1 [Reference]		1 [Reference]		1 [Reference]		1 [Reference]		1 [Reference]	
**Education level**														
Bachelor	14.8 (1.74-126.9)	.01	2.32 (0.49-10.8)	.28	7.71 (0.90-65.5)	.06	12.8 (1.50-109.3)	.02	29.1 (5.44-156.0)	.00	0.67 (0.14-3.14)	.62	1.28 (0.23-6.88)	.77
Master	13.2 (1.53-113.8)	.01	1.87 (0.39-8.85)	.42	6.00 (0.69-51.4)	.10	14.3 (1.66-124.0)	.01	23.5 (4.26-129.9)	.00	0.63 (0.13-2.97)	.56	1.54 (0.28-8.49)	.61
Doctor/ Ph.D.	5.18 (0.60-44.3)	.13	0.99 (0.21-4.61)	.99	3.46 (0.40-29.6)	.25	4.69 (0.54-40.1)	.15	5.79 (1.21-27.7)	.02	1.19 (0.25-5.54)	.82	0.69 (0.12-3.72)	.66
Other	1 [Reference]		1 [Reference]		1 [Reference]		1 [Reference]		1 [Reference]		1 [Reference]		1 [Reference]	
**Area of study**														
Arts & Humanities	19.5 (3.31-115.3)	.00	19.5 (3.31-115.3)	<.001	8.50 (1.40-51.4)	.02	13.3 (2.45-72.4)	.00	0.33 (0.02-4.09)	.39	0.23 (0.05-1.03)	.05	6.28 (1.29-30.5)	.02

Medicine	5.07 (1.28-20.1)	.02	7.52 (1.89-29.9)	.00	3.94 (0.79-19.6)	.09	5.07 (1.28-20.1)	.02	0.32 (0.03-2.90)	.31	0.74 (0.24-2.27)	.60	1.48 (0.48-4.52)	.49
Engineer	13.6 (3.75-49.2)	.00	15.1 (4.17-54.9)	.00	12.6 (2.82-56.9)	<.001	14.5 (4.02-52.9)	.00	0.73 (0.08-6.07)	.77	0.22 (0.08-0.62)	.00	7.64 (2.74-21.3)	.00
Agricultural	5.72 (1.50-21.8)	.01	8.88 (2.31-34.1)	<.001	7.36 (1.55-34.9)	.01	4.62 (1.21-17.6)	.02	0.16 (0.02-1.36)	.09	0.72 (0.24-2.11)	.55	4.28 (1.43-12.8)	.00
Business studies	10.2 (2.52-41.7)	<.001	17.1 (4.06-72.6)	.00	7.65 (1.54-37.7)	.01	8.17 (2.02-32.9)	.00	0.64 (0.06-6.68)	.71	0.42 (0.13-1.31)	.13	4.80 (1.47-15.6)	.00
Social Sciences and Law	15.4 (3.62-65.5)	.00	18.0 (4.16-77.8)	.00	11.3 (2.26-56.7)	.00	15.4 (3.62-65.5)	.00	0.91 (0.07-10.8)	.94	0.26 (0.08-0.86)	.02	13.2 (3.28-53.7)	.00
Language	7.33 (1.91-28.0)	.00	13.6 (3.50-53.3)	.00	4.95 (1.04-23.6)	.04	6.35 (1.66-24.2)	.00	1.52 (0.13-17.8)	.73	0.19 (0.06-0.57)	.00	4.80 (1.59-14.4)	.00
Other	1 [Reference]		1 [Reference]		1 [Reference]		1 [Reference]		1 [Reference]		1 [Reference]		1 [Reference]	
**Stay period in China (years)**														
<1	0.65 (0.30-1.41)	.28	0.61 (0.28-1.30)	.20	0.47 (0.18-1.23)	.12	1.05 (0.49-2.24)	.89	0.83 (0.31-2.26)	.72	1.82 (0.86-3.88)	.11	1.13 (0.53-2.40)	.75
<2	6.62 (3.74-11.7)	.00	6.21 (3.39-11.4)	.00	4.60 (2.76-7.66)	.00	7.49 (4.27-13.1)	.00	3.09 (1.27-7.55)	.01	0.35 (0.21-0.60)	.00	5.87 (3.12-11.0)	.00
2-3	2.63 (1.52-4.54)	<.001	3.15 (1.75-5.66)	.00	3.33 (1.92-5.75)	.00	3.67 (2.10-6.41)	.00	2.51 (0.97-6.49)	.05	0.77 (0.45-1.31)	.33	3.13 (1.70-5.78)	.00
>3	1 [Reference]		1 [Reference]		1 [Reference]		1 [Reference]		1 [Reference]		1 [Reference]		1 [Reference]	

Abbreviation: COR, Crude odds ratio; CI, confidence interval; NA, not applicable.

Supplement Table S2. Risk Factors for Psychological Outcomes Identified by Binary Logistic Regression Analysis (multivariate analysis).

	Depression		Anxiety		Stress		Insomnia		Psychological Distress		Loneliness		Fear	
**Variables**	**AOR (95% CI)**	***P* value**	**AOR (95% CI)**	***P* value**	**AOR (95% CI)**	***P* value**	**AOR (95% CI)**	***P* value**	**AOR (95% CI)**	***P* value**	**AOR (95% CI)**	***P* value**	**AOR (95% CI)**	***P* value**
**Gender**														
Male	1.66 (0.73-3.75)	.22	1.68 (0.76-3.73)	.19	1.07 (0.49-2.34)	.85	2.10 (0.91-4.86)	.08	1.17 (0.36-3.82)	.78	0.61 (0.30-1.24)	.18	1.66 (0.79-3.50)	.17
Female	1 [Reference]		1 [Reference]		1 [Reference]		1 [Reference]		1 [Reference]		1 [Reference]		1 [Reference]	
**Age (years)**														
18-25	1.46 (0.32-6.54)	.61	1.30 (0.31-5.45)	.71	1.47 (0.29-7.31)	.63	2.52 (0.50-12.5)	.25	0.42 (0.05-3.22)	.41	0.63 (0.16-2.35)	.49	0.64 (0.15-2.66)	.54
26-30	2.75 (0.76-9.95)	.12	3.17 (0.93-10.7)	.06	1.60 (0.37-6.85)	.52	3.57 (0.85-14.9)	.08	1.78 (0.43-7.35)	.42	0.88 (0.27-2.83)	.83	1.57 (0.48-5.16)	.45
31-35	4.28 (1.33-13.6)	.01	6.65 (2.11-20.9)	<.001	4.90 (1.32-18.1)	.01	7.58 (2.01-28.6)	.00	4.95 (1.33-18.4)	.01	0.42 (0.14-1.22)	.11	2.73 (0.92-8.11)	.07
36-40	1 [Reference]		1 [Reference]		1 [Reference]		1 [Reference]		1 [Reference]		1 [Reference]		1 [Reference]	
**Geographical regions, World**														
Asia	1.41 (0.07-28.1)	.82	NA	NA	2.56 (0.18-35.3)	.48	4.60 (0.23-91.9)	.31	6.96 (0.20-241.1)	.28	0.13 (0.01-1.99)	.14	20.8 (1.45-299.0)	.02
Africa	0.86 (0.03-19.5)	.92	NA	NA	7.05 (0.40-122.2)	.17	2.13 (0.08-51.3)	.64	3.40 (0.08-135.9)	.51	0.23 (0.01-3.78)	.30	15.5 (0.94-255.5)	.05
Europe	5.09 (0.12-214.7)	.39	NA	NA	1.56 (0.06-37.6)	.78	2.96 (0.08-109.9)	.55	1.98 (0.03-132.0)	.74	0.15 (0.007-3.60)	.24	4.19 (0.17-98.3)	.37
America	1 [Reference]		1 [Reference]		1 [Reference]		1 [Reference]		1 [Reference]		1 [Reference]		1 [Reference]	
**Geographical regions, China**														
Eastern	0.24 (0.03-1.59)	.14	0.13 (0.01-1.20)	.07	0.55 (0.12-2.44)	.44	0.07 (0.01-0.54)	.01	0.82 (0.03-18.6)	.90	0.76 (0.25-2.28)	.63	0.66 (0.08-5.29)	.70
Northern	0.21 (0.02-1.82)	.15	0.40 (0.03-4.63)	.46	1.01 (0.18-5.62)	.99	0.30 (0.03-3.14)	.31	0.37 (0.01-11.3)	.57	0.92 (0.23-3.65)	.91	0.47 (0.05-4.49)	.51
Southwest	0.21 (0.04-1.06)	.05	0.18 (0.02-1.26)	.08	0.48 (0.14-1.68)	.25	0.10 (0.01-0.59)	.01	0.27 (0.02-3.04)	.29	0.55 (0.14-2.05)	.37	0.20 (0.04-1.04)	.05
Northeast	0.12 (0.01-1.01)	.05	0.11 (0.01-1.25)	.07	0.57 (0.09-3.49)	.54	0.09 (0.01-0.87)	.03	0.25 (0.01-4.96)	.36	0.39 (0.07-1.96)	.25	1.31 (0.08-20.2)	.84
Central	0.81 (0.12-5.10)	.82	0.95 (0.10-8.54)	.96	2.43 (0.56-10.5)	.23	0.42 (0.06-2.94)	.38	NA	NA	0.15 (0.03-0.63)	.01	0.31 (0.04-2.08)	.23
Southern	0.70 (0.10-4.59)	.71	0.51 (0.05-4.48)	.54	1.06 (0.25-4.49)	.93	0.46 (0.06-3.53)	.46	NA	NA	0.11 (0.01-0.81)	.03	1.22 (0.15-9.71)	.84
Northwest	1		1		1		1		1		1		1	

	[Reference]		[Reference]		[Reference]		[Reference]		[Reference]		[Reference]		[Reference]	
**Religion**														
Islam	0.42 (0.02-6.88)	.54	NA	NA	NA	NA	0.51 (0.03-8.42)	.64	NA	NA	1.17 (0.09-14.8)	.90	1.23 (0.08-17.9)	.87
Hinduism	0.13 (0.007-2.75)	.19	NA	NA	NA	NA	0.26 (0.01-5.56)	.39	NA	NA	0.22 (0.01-3.85)	.30	0.81 (0.04-15.1)	.89
Buddhist	0.64 (0.03-12.1)	.76	NA	NA	NA	NA	1.00 (0.05-19.7)	.99	NA	NA	0.55 (0.03-8.17)	.66	1.59 (0.09-27.1)	.74
Christian	1.54 (0.07-31.8)	.78	NA	NA	NA	NA	0.49 (0.02-10.4)	.65	NA	NA	0.85 (0.05-12.9)	.90	3.47 (0.18-63.9)	.40
Others	NA	NA	NA	NA	NA	NA	NA	NA	NA	NA	NA	NA	NA	NA
No religion	1 [Reference]		1 [Reference]		1 [Reference]		1 [Reference]		1 [Reference]		1 [Reference]		1 [Reference]	
**Marital status**														
Unmarried	NA	NA	NA	NA	NA	NA	NA	NA	NA	NA	NA	NA	NA	NA
Married	NA	NA	NA	NA	NA	NA	NA	NA	NA	NA	NA	NA	NA	NA
Divorced/Separated/ Widowed	1 [Reference]		1 [Reference]		1 [Reference]		1 [Reference]		1 [Reference]		1 [Reference]		1 [Reference]	
**Living conditions**														
Alone	NA	NA	NA	NA	NA	NA	NA	NA	NA	NA	NA	NA	NA	NA
Roommate	NA	NA	NA	NA	NA	NA	NA	NA	NA	NA	NA	NA	NA	NA
Family	NA	NA	NA	NA	NA	NA	NA	NA	NA	NA	NA	NA	NA	NA
Other	1 [Reference]		1 [Reference]		1 [Reference]		1 [Reference]		1 [Reference]		1 [Reference]		1 [Reference]	
**Living place**														
Dormitory	1.62 (0.68-3.85)	.27	0.86 (0.35-2.09)	.75	1.37 (0.56-3.34)	.48	0.73 (0.31-1.75)	.49	NA	NA	1.28 (0.59-2.76)	.53	1.17 (0.49-2.80)	.71
Hotel	0.47 (0.03-6.63)	.58	0.66 (0.09-4.54)	.67	1.67 (0.14-19.3)	.68	1.51 (0.15-14.3)	.71	NA	NA	0.32 (0.04-2.52)	.28	1.75 (0.28-10.6)	.54
Outside of the campus	1 [Reference]		1 [Reference]		1 [Reference]		1 [Reference]		1 [Reference]		1 [Reference]		1 [Reference]	
**Education level**														
Bachelor	7.87 (0.62-98.8)	.11	0.43 (0.05-3.21)	.41	2.78 (0.22-34.3)	.42	6.22 (0.46-83.1)	.16	384.4 (13.6-108.1)	.00	2.66 (0.39-17.8)	.31	1.16 (0.14-9.04)	.88
Master	5.66 (0.41-77.3)	.19	0.25 (0.03-1.87)	.17	1.58 (0.12-21.0)	.72	8.73 (0.59-129.1)	.11	252.3 (10.4-607.3)	<.001	1.13 (0.17-7.57)	.89	1.12 (0.14-8.65)	.91
Doctor/ Ph.D.	6.26 (0.43-90.2)	.17	0.41 (0.05-2.94)	.37	2.36 (0.16-33.1)	.52	10.4 (0.65-165.2)	.09	66.9 (3.22-139.3)	.00	0.76 (0.11-5.08)	.78	0.98 (0.13-7.32)	.98
Other	1 [Reference]		1 [Reference]		1 [Reference]		1 [Reference]		1 [Reference]		1 [Reference]		1 [Reference]	
**Area of study**														
Arts & Humanities	18.0 (0.02-1.09)	.00	24.4 (3.02-197.2)	.00	7.01 (0.88-55.3)	.06	9.59 (1.30-70.5)	.02	0.08 (0.004-1.95)	.12	0.30 (0.05-1.59)	.15	4.00 (0.66-24.2)	.13

Medicine	5.63 (0.05-0.93)	.03	11.8 (2.39-58.7)	.00	3.61 (0.59-22.0)	.16	7.08 (1.39-35.8)	.01	0.16 (0.01-2.81)	.21	1.08 (0.31-3.82)	.89	1.68 (0.45-6.28)	.43
Engineer	5.92 (0.05-0.71)	.02	7.03 (1.59-30.9)	.01	5.29 (0.96-28.9)	.05	6.53 (1.43-29.6)	.01	0.19 (0.01-2.98)	.23	0.43 (0.14-1.34)	.15	4.30 (1.28-14.4)	.01
Agricultural	5.43 (0.04-0.79)	.03	10.2 (2.11-49.5)	.00	5.89 (0.99-35.0)	.05	3.05 (0.62-15.0)	.17	0.06 (0.004-0.90)	.04	1.17 (0.35-3.91)	.79	3.22 (0.90-11.5)	.07
Business studies	13.9 (0.01-0.40)	.00	33.6 (5.72-198.3)	.00	7.17 (1.06-48.5)	.04	10.7 (1.88-61.2)	.00	0.29 (0.01-5.77)	.42	0.45 (0.12-1.66)	.23	6.50 (1.52-27.7)	.01
Social Sciences and Law	15.3 (0.04-1.39)	.00	11.4 (1.93-67.0)	.00	5.30 (0.83-33.7)	.07	9.70 (1.57-59.8)	.01	1.59 (0.04-57.9)	.79	0.50 (0.12-1.94)	.31	13.5 (2.37-77.3)	.00
Language	10.2 (0.02-0.66)	.01	37.8 (5.85-245.1)	.00	3.22 (0.48-21.5)	.22	8.55 (1.46-49.9)	.01	0.49 (0.01-17.1)	.69	0.17 (0.04-0.70)	.01	8.81 (1.84-42.1)	.00
Other	1 [Reference]		1 [Reference]		1 [Reference]		1 [Reference]		1 [Reference]		1 [Reference]		1 [Reference]	
**Stay period in China (years)**														
<1	0.34 (0.11-1.01)	.05	0.37 (0.12-1.11)	.07	0.32 (0.09-1.07)	.06	0.82 (0.28-2.35)	.71	0.50 (0.11-2.28)	.37	3.37 (1.29-8.80)	.01	0.70 (0.25-1.92)	.49
<2	3.92 (1.70-9.06)	<.001	6.49 (2.69-15.6)	.00	2.51 (1.15-5.48)	.02	4.49 (1.93-10.4)	.00	1.27 (0.35-4.58)	.71	0.61 (0.29-1.26)	.18	4.67 (1.93-11.3)	<.001
2-3	1.23 (0.58-2.61)	.58	2.93 (1.32-6.51)	.00	2.45 (1.17-5.14)	.01	1.97 (0.91-4.27)	.08	1.02 (0.31-3.35)	.97	0.93 (0.47-1.85)	.85	1.68 (0.76-3.72)	.19
>3	1 [Reference]		1 [Reference]		1 [Reference]		1 [Reference]		1 [Reference]		1 [Reference]		1 [Reference]	

Abbreviation: AOR, adjusted odds ratio; CI, confidence interval; NA, not applicable.
